# Noninvasive fractional flow reserve derived from coronary computed tomography angiography for identification of ischemic lesions: a systematic review and meta-analysis

**DOI:** 10.1038/srep29409

**Published:** 2016-07-05

**Authors:** Wen Wu, Dao-Rong Pan, Nicolas Foin, Si Pang, Peng Ye, Niels Holm, Xiao-Min Ren, Jie Luo, Aravinda Nanjundappa, Shao-Liang Chen

**Affiliations:** 1 Department of Cardiology, Nanjing First Hospital, Nanjing Medical University, Nanjing, 210006, P.R. China; 2 National Heart Research Institute, National Heart Centre Singapore, 169609, Singapore; 3 Department of Cardiology, Zhongda Hospital, Medical School of Southeast University, Nanjing 210009, China; 4 Department of Cardiology, Aarhus University Hospital, Aarhus, 8000, Denmark; 5 Division of Vascular Surgery, West Virginia University, Morgantown, 25304, USA

## Abstract

Detection of coronary ischemic lesions by fractional flow reserve (FFR) has been established as the gold standard. In recent years, novel computer based methods have emerged and they can provide simulation of FFR using coronary artery images acquired from coronary computed tomography angiography (FFR_CT_). This meta-analysis aimed to evaluate diagnostic performance of FFR_CT_ using FFR as the reference standard. Databases of PubMed, Cochrane Library, EMBASE, Medion and Web of Science were searched. Seven studies met the inclusion criteria, including 833 stable patients (1377 vessels or lesions) with suspected or known coronary artery disease (CAD). The patient-based analysis showed pooled estimates of sensitivity, specificity and diagnostic odds ratio (DOR) for detection of ischemic lesions were 0.89 [95%confidence interval (CI), 0.85–0.93], 0.76 (95%CI, 0.64–0.84) and 26.21 (95%CI, 13.14–52.28). At a per-vessel or per-lesion level, the pooled estimates were as follows: sensitivity 0.84 (95%CI, 0.80–0.87), specificity 0.76 (95%CI, 0.67–0.83) and DOR 16.87 (95%CI, 9.41–30.25). Area under summary receiver operating curves was 0.90 (95%CI, 0.87–0.92) and 0.86 (95%CI, 0.83–0.89) at the two analysis levels, respectively. In conclusion, FFR_CT_ technology achieves a moderate diagnostic performance for noninvasive identification of ischemic lesions in stable patients with suspected or known CAD in comparison to invasive FFR measurement.

As the most common cause of cardiovascular disease mortality, the prevalence of coronary artery disease (CAD) is still increasing worldwide^1^. For diagnosis of CAD, invasive coronary angiography (ICA), the standard approach historically, is limited to provide only anatomic information^2^. Percutaneous coronary intervention (PCI) decision based merely on ICA can result in unbenefited stenting of functionally non-significant lesions or inappropriate deferral of PCI of functionally significant lesions^3^. Fractional flow reserve (FFR), measured during ICA, has been established as the reference standard in evaluating the functional significance of a coronary stenosis^4^. In addition, the clinical utility of FFR as a decisive tool for revascularization therapy has been evaluated by several prospective randomized trials, demonstrating how FFR-guided PCI can optimize benefits of revascularization and improve long-term outcomes compared with angiographic guidance alone^5^,^6^,^7^,^8^. Revascularization decision with FFR guidance has also been shown to be a sound strategy in terms of cost-benefits with significantly fewer stents implanted and less contrast agent used in comparison with PCI guided by ICA alone^9^. Nevertheless, FFR is an invasive method after all, bringing potential procedural risks for patients^4^.

An alternative technique called noninvasive fractional flow reserve derived from coronary computed tomography angiography (FFR_CT_) has been developed^10^. Through utilization of computational fluid dynamics and coronary artery images acquired from coronary computed tomography angiography, FFR_CT_ enables estimation of FFR value without the need for additional invasive imaging, modification of acquisition protocols, or extra administration of medication. Hence, it is able to provide information both on the anatomic severity of a coronary lesion and its functional significance in a relatively safe and economical manner. Since its feasibility was initially validated in 2011^11^, a number of clinical studies have been conducted to evaluate the diagnostic efficacy of FFR_CT_ using FFR as the reference standard^12^,^13^,^14^,^15^.

Previous meta-analyses have evaluated the diagnostic performance of FFR_CT_ both at the per-patient level and the per-vessel or per-lesion level as defined by the invasive FFR^16^,^17^. However, concerns have been raised about the applicability of univariate model in pooling estimates of sensitivity and specificity, either with fixed- or random-effects model, which might inadvertently produce inaccurate results by ignoring threshold effects and correlation between the two estimates^18^. Moreover, results of new diagnostic accuracy tests for assessment of FFR_CT_ have recently been published as full papers^19^,^20^. Therefore, an updated meta-analysis was carried out to comprehensively search and review evidence available heretofore and derive reliable assessment of the diagnostic performances of FFR_CT_ using a bivariate model as the method for pooling diagnostic measures.

## Results

### Literature search and characteristics of the included studies

**Figure 1:**
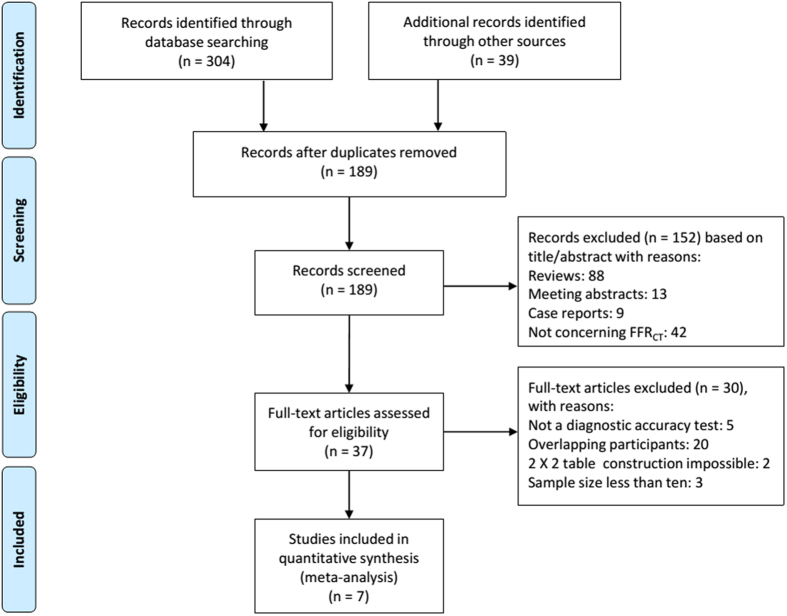
Flow chart of search and selection of eligible studies. Abbreviations: n, number of studies; FFR_CT_: fractional flow reserve derived from computed tomography.

The initial search yielded 343 items from PubMed, the Cochrane Library, EMBASE, Medion and Web of Science. Because of using a board search strategy, most of the results were not eligible. After exclusion based on title, abstract and full-text, seven eligible studies fulfilled the pre-defined inclusion criteria and were included in this systematic review and meta-analysis^11^,^12^,^13^,^14^,^15^,^19^,^20^. Procedure of studies inclusion was described in Fig. 1. Characteristics of included studies and patients’ baseline demographic were presented in Table 1. Inclusion and exclusion criteria of each study were listed in Supplementary Table 1.

Out of these seven studies published between 2011 and 2015, four were prospective multicenter trials^11^,^12^,^13^,^15^, and the remaining three were retrospective single-center trials^14^,^19^,^20^. A total of 833 patients (1377 vessels or lesions) were analyzed. The sample size of each study ranged from 21 to 254 patients (23 to 484 vessels or lesions). All participants were stable patients with suspected or known CAD. More males were included than females, with mean age ranging from 60 to 65 years, diabetes prevalence from 14% to 32% in each study. All CT images in the seven studies were acquired in accordance with a standard coronary computed tomography angiography (CCTA) protocol using multi-slice CT scanner (64 slices or higher) or dual source CT scanner. For exact comparison, the point of FFR_CT_ estimation was deduced from the position of the pressure guide wire for invasive FFR. And the process of FFR_CT_ calculation was performed using either a HeartFlow or a Siemens software. In terms of cut-off points, most studies used the same one to define positive results (FFR_CT_ ≤ 0.80; FFR ≤ 0.80) except one by Renker *et al*. (FFR_CT_ < 0.80; FFR < 0.80). The diagnostic performance of FFR_CT_ at the per-vessel or per-lesion level were reported by all included studies^11^,^12^,^13^,^14^,^15^,^19^,^20^, while the per-patient analysis was pooled from five studies^11^,^12^,^14^,^15^,^20^. The count data on the per-patient and the per-vessel or per-lesion basis for primary study including true positive (TP), false positive (FP), false negative (FN) and true negative (TN) were extracted and shown in Table 1.

### Quality of studies

The results of Quality Assessment of Diagnostic Accuracy Studies (QUADAS-2) for included studies were summarized in Table 2 and Supplementary Fig. 1. In terms of signaling questions about interval between index and reference test in flow and timing of reference standard, most studies were recorded as “unclear” apart from the DISCOVER-FLOW trial. Nevertheless, all studies were well rated as “low risk” in all domains in relation with risk bias and applicability concerns. Proportion of studies with low risk of bias and with low concerns regarding applicability were 100%, respectively. Hence, all these primary diagnostic accuracy tests were included for synthesis of diagnostic performance.

### Synthesis of diagnostic performance

Pooled diagnostic performances of FFR_CT_ at the two analysis levels were summarized in Table 3.

**Figure 2:**
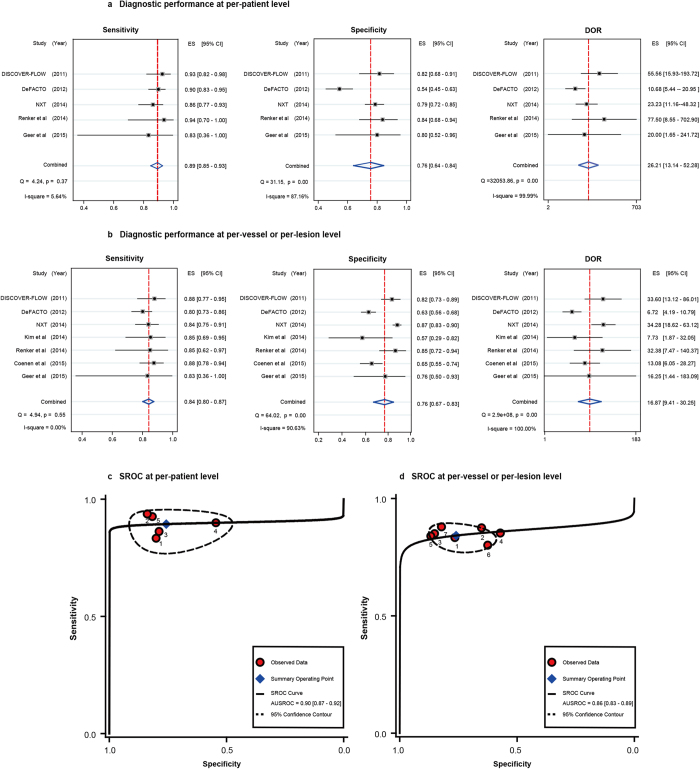
Combined diagnostic performances of FFR_CT_ at the per-patient level and at the per- vessel or per-lesion level. Abbreviations: AUSROC, area under summary receiver operating curve; DOR, diagnostic odds ratio; ES, estimates; NLR, negative likelihood ratio; PLR, positive likelihood ratio; SROC, summary receiver operating curve.

At the per-patient level, the pooled estimates of sensitivity, specificity and diagnostic odds ratio (DOR) for the detection of ischemia-causing coronary stenosis were 0.89 [95% confidence interval (CI), 0.85–0.93], 0.76 (95% CI, 0.64–0.84) and 26.21 (95% CI, 13.14–52.28) (Fig. 2a). The corresponding summary positive likelihood ratio (PLR) and negative likelihood ratio (NLR) were 3.68 (95% CI, 2.41–5.61) and 0.14 (95% CI, 0.09–0.21), respectively, and the combined diagnostic score was 3.27 (95% CI, 2.58–3.96) (Table 3).

The pooled estimates of diagnostic performance at the per-vessel or per-lesion level were as follows: sensitivity 0.84 (95% CI, 0.80–0.87), specificity 0.76 (95% CI, 0.67–0.83), DOR 16.87 (95%CI, 9.41–30.25), PLR 3.51 (95% CI, 2.44–5.03), NLR 0.21 (95% CI, 0.16–0.27) and diagnostic score 2.83 (95% CI, 2.24–3.41) (Fig. 2b and Table 3).

The area under summary receiver operating curves (SROC) at the per-patient level and at the per-vessel or per lesion level was 0.90 (95% CI, 0.87–0.92) and 0.86 (0.83–0.89), respectively (Fig. 2c,d). Through graphical examination of SROC plot, the threshold effects were deemed absent either at the per patient level nor at the per-vessel or per-lesion level, with Spearman correlation coefficients being−0.60 (P = 0.285) and−0.036 (P = 0.939) respectively.

**Figure 3:**
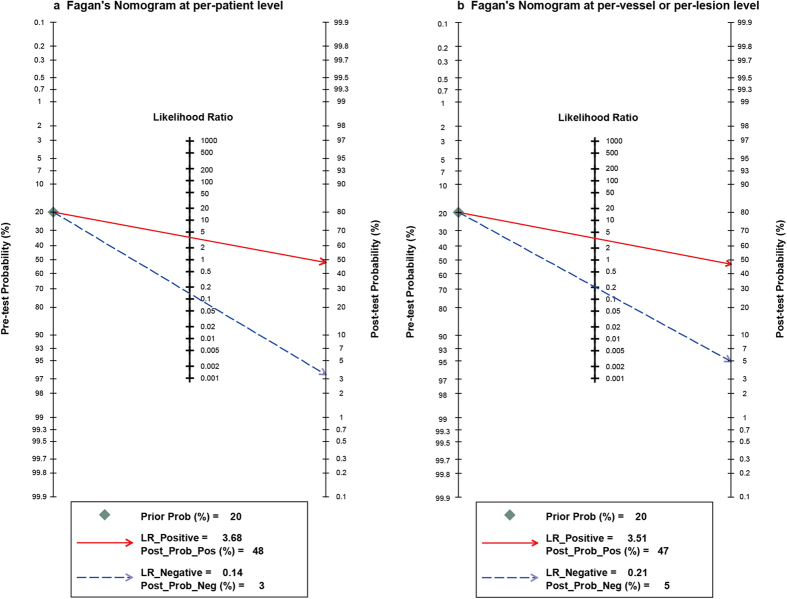
Fagan’s Nomogram plot analysis to evaluate the clinical utility of FFR_CT_ for the detection of ischemic lesions using FFR as reference standard. In each plot, a vertical axis on the left showed the fixed pre-test probability. Using the likelihood ratio in the middle axis, post-test probability (patient’s probability of having the disease after the index test result was known) was acquired. (**a**) With a pre-test probability of a positive FFR of 20%, the post-test probability of positive FFR, given positive and negative FFR_CT_ results, were 48% and 3%. (**b**) With a pre-test probability of a positive FFR of 20% on a per-lesion basis, the post-test probability of FFR, given positive and negative FFR_CT_ results, were 47% and 5%.

Fagan’s Nomogram analysis (Fig. 3) revealed that, with fixed pre-test probability of 20% and a pooled PLR of 3.68 at the per-patient level and 3.51 at the per-vessel or per-lesion level, the post-test probability was increased to 48% and to 47% respectively. Conversely, with a combined NLR of 0.14 and 0.21, the post-test probability was decreased to 3% and 5% at the two analysis levels, respectively.

### Meta-regression analysis

The meta-regression analysis at the per-patient level and at the per-vessel or per-lesion level indicated that the publication date, study design, sample size, age, gender, diabetes, CT scanner type, time period between FFR_CT_ and FFR, software for FFR_CT_ calculation and cut-off value for FFR_CT_ and FFR were not sources of heterogeneity.

### Publication bias

**Figure 4:**
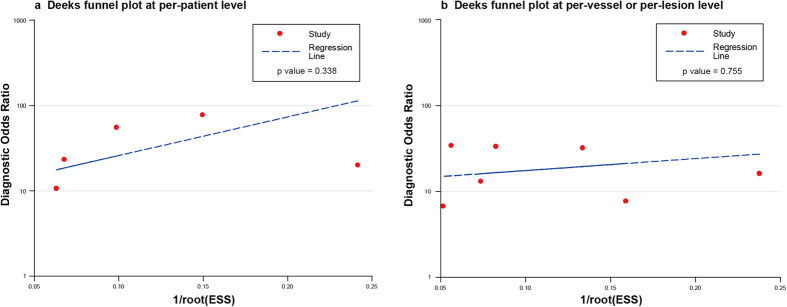
Deeks funnel plot for detecting publication bias. Abbreviations: ESS, effective sample size.

The publication bias, at first, was visually assessed using Deeks funnel plots (Fig. 4). The two plots were both in symmetrical funnel shape, indicating the publication bias was likely absent. Moreover, P values for the Deeks funnel plot asymmetry test on the per-patient basis and on the per-vessel or per-lesion basis were 0.34 and 0.76, respectively. Therefore, publication bias did not exist at the two analysis levels.

## Discussion

This current systematic review and meta-analysis of seven diagnostic accuracy studies including 833 suspected or known CAD patients (1377 vessels or lesions) investigated the potential of FFR_CT_ for identification of ischemic lesions. Recently approved by the US Food and Drug Administration, FFR_CT_ based on computational fluid dynamics allows for virtual assessment of “3-vessel FFR” at any point within the coronary artery bed from typically acquired CCTA imaging^21^. Thus, not only the anatomic severity but also the estimated physiological significance of coronary stenosis was taken into account. Notably, FFR_CT_ technology is moving forward from comparison with invasive FFR measures and the real-world implementation of FFR_CT_ for clinical decision making has been validated^22^.

As widely known, CCTA as a noninvasive technique for evaluation of CAD has been rapidly progressing in recent years^23^. And high diagnostic accuracy of 64-, 128-, 256-, 320-slice, CCTA in identification of CAD has been demonstrated by multiple studies^24^,^25^,^26^,^27^. In particular, the constantly high negative predictive value (NPV) makes CCTA a “gate keeper” in ruling out significant CAD. Indeed, for detection of ischemic lesions confirmed by the invasive FFR, CCTA maintained high combined estimates of sensitivity and NPV (92% and 87%, respectively) while the specificity and positive predictive value (PPV) was only modest (43% and 57%, respectively)^17^. As recently reported by an integrated analysis of three pivotal trials (DISCOVER-FLOW, DeFACTO and NTX), FFR_CT_ exhibited numerically higher sensitivity (86.1% vs 82.8%, P = 0.369) and NPV (92.5% vs 91.6%, P = 0.645)^28^. Besides, FFR_CT_ was found with significantly improved specificity (77.7% vs 55.7%, P < 0.001) and PPV (60.8% vs 38.7%, P < 0.001) compared with CCTA. In the current study, the pooled estimates of sensitivity (0.89 at the per-patient level and 0.84 at the per-vessel or per-lesion level) and specificity (0.76 at both of the two levels) was in line with the former findings^16^,^17^,^28^. To evaluate the real-world performance of FFR_CT_ with improved specificity in differentiating a true-positive from a false-positive test, a pragmatic comparative effectiveness PLATFORM (Prospective Longitudinal Trial of FFR_CT_: Outcomes and Resource Impacts) trial was conducted^29^. Among patients referred to ICA in this trial, 12% of patients in the arm of FFR_CT_ combined with CCTA were found with no obstructive CAD confirmed by ICA, while in the ICA arm the rate was 73% (risk difference 61%, P < 0.0001). In a recent report of PLATFORM trial regarding economic implications of FFR_CT_ strategy, patients in the arm of FFR_CT_ and CCTA were associated with a 32% reduction in costs compared with patients in the ICA arm^30^. This encouraging outcome which stemmed from a significant reduction in false-positive noninvasive test results was believed to be of considerable importance for health care milieu.

The area under the SROC provides a general evaluation of diagnostic efficacy. According to the recommended guideline for interpretation of AUSROC value^31^, the diagnostic ability of FFR_CT_ for identification of ischemic lesions was moderate at the per-patient level and at the per-vessel or per-lesion level (AUSROC: 0.90, 0.86). DOR is a measure combining sensitivity and specificity together. And the higher value of DOR, the higher the diagnostic efficacy of the test to detect between subjects with and without the disease of interest. Our pooled estimates of DOR were 26.21 and 16.87 at the two analysis levels, respectively, indicating a moderate diagnostic value of FFR_CT_ in detection of lesion-specific ischemia. As shown in Fig. 4, Fagan’s nomogram revealed that the application of FFR_CT_ would raise the post-test probability of patients having ischemic lesions (confirmed by invasive FFR) from 20% to 48% at the per-patient level and to 47% at the per-vessel or per-lesion level. Meanwhile, the probability of having negative FFR status was decreased from 20% to 3% on the per-patient basis and to 5% on the per-vessel or per-lesion basis. According to these findings, FFR_CT_ was demonstrated to be of moderate value in determining the lesions causing ischemia.

It is generally known that CCTA image quality could be suboptimal owing to technical issues such as severe calcification^23^. In this situation, CCTA examination was uninterpretable and the subsequent FFR_CT_ analysis could not be done. 12.05% of subjects (5.21% with severe calcification) in the NXT trial and 8.20% of subjects (4.92% with severe calcification) in the study by Coenen *et al*. were excluded from analysis due to unavailable CCTA image. Hence, there were concerns about the influence of selection bias and test heterogeneity in the pooled diagnostic accuracy of FFR_CT_. However, according to a sub-study of NXT trial^32^, FFR_CT_ was immune to the influence of calcification since no difference of diagnostic efficacy was observed across any coronary artery calcium (CAC) score quartiles. Even among patients with Agatston CAC score above 416, FFR_CT_ was observed with improved detection of ischemic lesions over the CCTA (AUC: 0.91 vs 0.71; P = 0.0004).

In addition to these diagnostic features, an emerging body of clinical evidence has indicated that the application of FFR_CT_ may have an influence on therapeutic strategy^21^. In the RIPCORD (Does Routine Pressure Wire Assessment Influence Management Strategy at Coronary Angiography for Diagnosis of Chest Pain) trial, 36% of patients were observed with a change in diagnostic and therapeutic management. Based on the results of FFR_CT_, 12% of patients with planned medical therapy alone were sent for ICA, while 30% of patient assigned with ICA were reassigned to medical therapy^33^. Among patients with intended revascularization, 18% were found to have ischemic lesions in different vessels. Furthermore, not merely diagnosing ischemic lesions before PCI, FFR_CT_ was also capable of predicting hemodynamic effect of revascularization, thus enabling “virtual stenting” of a coronary lesion, which may be beneficial to treatment planning before invasive procedures^13^.

Compared with high diagnostic performance of FFR_CT_ for identification of lesion-specific ischemia reported by the most recent meta-analysis^16^, the diagnostic efficacy was moderate in the present study. The difference can be explained by several reasons. Firstly, the current meta-analysis retrieved studies until January 31st, 2016, adding two studies with 127 patients and 212 vessels or lesions, so that more comprehensive evaluation of the diagnostic efficacy of FFR_CT_ was obtained^19^,^20^. Secondly, software for FFR_CT_ analysis used in three studies was different from the rest. Last but not least, a different statistical method for combining diagnostic estimates was applied in the current study.

Combined with patient-specific CT image-based modeling, computational fluid dynamics technology and lumped cardiac and coronary microcirculatory model, FFR_CT_ is applied for determining the individual physiologic conditions of CAD patients^34^. Although two different kinds of software, HeartFlow and Siemens cFFR, were used in the included studies based on the same principles mentioned above, the algorithms for FFR_CT_ analysis were different. In terms of HeartFlow^11^,^12^,^13^,^15^, the CCTA data were transferred to a parallel supercomputer off-site for construction of the coronary flow model on the basis of full order algorithm. Meanwhile, FFR_CT_ calculation in studies with application of Siemens cFFR^14^,^19^,^20^ was based on integration of the full and reduced order algorithm in the software. In atherosclerotic or stenotic vascular segments of interest, the full order algorithm was applied while the reduced order algorithm, usually performed on a regular workstation on-site within hospital, was used for the rest healthy coronary artery segments.

Different from therapeutic or intervention studies, diagnostic accuracy studies are characteristic of evaluation by a pair of summary estimates (sensitivity and specificity), different thresholds chosen from a wide range of values, and commonly larger between-study heterogeneity^31^. Bivariate model is a multivariate method, jointly analyzing and assessing diagnostic accuracy of a test^35^. This model, unlike separate pooling methods such as fixed- or random-effects model, utilizes a hierarchical structure of the distribution of data in terms of two levels. In detail, a within-study variability is considered by supposing a binomial distribution for the sensitivity and 1-specificity of each study at the first level, while at the second level a between-study heterogeneity is modeled by assuming a bivariate normal distribution through the logit-transformed sensitivity and specificity^18^. Hence, bivariate model preserves the two-dimensional nature of diagnostic accuracy, allows for a potential correlation between sensitivity and specificity, and manages the differences in the precision of the two estimates. It should be noted that, the bivariate model is currently regarded as the optimal method for acquiring pooled statistics from diagnostic accuracy trials with recommendation from the Diagnostic Test Accuracy Working Group of the Cochrane Collaboration and the Agency for Healthcare Research and Quality (AHRQ)^31^. As shown in Supplementary Table 2, diagnostic performances of FFR_CT_ at the two analysis levels with or without two recent studies (Coenen *et al*. and Geer *et al*.^19^,^20^) using two different pooling statistical methods was pooled. The synthesized AUSROC by the bivariate model was numerically conservative in comparison with that by univariate model both in per-patient level and per-vessel or per-lesion level. In consideration of the significant between-study heterogeneity in this study, it is appropriate to use the model of bivariate. Therefore, the pooled estimates of FFR_CT_ diagnostic measures in our systematic review and meta-analysis, with incorporation of updated clinical data and application of the optimal combing method, were believed to be comprehensive and reliable.

There are several limitations which deserve to be mentioned. Firstly, the number of studies included in this systematic review and meta-analysis was relatively small. Secondly, subjects in our study were limited to stable patients with stable known or suspected CAD, excluding patients with complex lesions and a history of prior percutaneous coronary intervention or coronary bypass surgery in vessels of interest. Hence the diagnostic performance of FFR_CT_ on wider groups of patients are unknown. Thirdly, diagnostic efficacy of FFR_CT_ in patients with intermediate lesions was reported by DeFACTO study and NXT study at the per-patient level^12^,^15^, while DISCOVER-FLOW study, DeFACTO study and study by Coenen *et al*. have reported diagnostic measures on the per-vessel or per-lesion basis^11^,^12^,^19^. In consideration of the minimum number of studies required for data synthesis using the bivariate model, the combination of diagnostic accuracy of FFR_CT_ in patients with coronary stenosis of intermediate severity was not performed. In addition, covariates that affect sensitivity and specificity in the bivariate model were not used due to limited data presented by the original included studies. Finally, heterogeneities did exist in this study but the sources of them could not be identified using the current pre-defined factors.

In conclusion, up-to-date evidence available about the FFR_CT_ diagnostic efficacy was synthesized in the present systematic review and meta-analysis with inclusion of seven diagnostic accuracy studies. And FFR_CT_ may in the future be of help for identification of lesions causing ischemia among stable patients with suspected or known CAD. However, more pragmatic clinical trials with larger subsets of CAD patients are warranted to demonstrate its clinical benefits in real world practice.

## Methods

### Literature search strategy

To identify eligible studies published through January 31st, 2016, the main search was conducted in databases of PubMed, the Cochrane Library, EMBASE, Medion and Web of Science using various combinations of Medical Subject Headings (MeSH) and non-MeSH terms: fractional flow reserve, FFR, noninvasive fractional flow reserve, noninvasive FFR, non-invasive fractional flow reserve, non-invasive FFR and FFR derived from coronary computed tomography angiography. We also searched related reports from major annual meetings in field of Cardiology and Radiology. Manual search of reference sections of eligible studies and all relevant reviews was also performed. We did not apply any language restrictions.

### Study Identification

Two investigators (Wen Wu and Dao-Rong Pan) independently carried out the systematic literature search, study identification, data extraction and quality assessment in accordance with Preferred Reporting Items for Systematic reviews and Meta-Analyses (PRISMA) statement^36^. (kappa index = 0.956, indicating excellent between the two investigators). Any discrepancy was resolved by discussion with a third reviewer (Si Pang). Studies were included when following criteria were met: (1) conducted as a diagnosis accuracy test; (2) the index test was FFR_CT_ and the standard test was FFR; (3) at least 10 study subjects were involved; (4) a two-by-two contingency table could be constructed from data presented by the study. Trials were excluded on the basis of the following criteria: (1) phantom-only study; (2) studies conducted on animals or *in-vitro* systems; (3) the article pertained to review, case report, editorial comment or prognostic study; (4) study containing overlapping participants. Notably, article by the same author or research group was included only when it used a different sample of patients.

### Data extraction

At first, basic information about studies including first author, study design and publication year was extracted. Further extracted variables comprising patients’ baseline demographics, technical information and absolute numbers of TP, FP, FP and FN were also exacted from eligible studies using a standardized form.

### Quality assessment

The QUADAS-2 tool was used to flag facets of a study which were associated with the potential bias^37^. Aimed to help reviewers judge risks of bias, QUADAS-2 consists of four key domains including patient selection, index test, reference test flow of patients through the study and timing of the index test and the reference test (flow and timing). These four domains were applied for assessing risks of bias and the first three were used to evaluate concerns of applicability. Based on reviewer authors’ answers for all signaling questions in each domain, risks of bias were judged as “low risk”, “high risk” or “unclear risk”. In terms of applicability concerns, review authors were asked to record relevant information and then to assess their concerns if the study matched the review question. In a similar way, concerns with respect to applicability were rated as “low risk”, “high risk” or “unclear risk”. And the results of the QUADAS-2, with numbers of studies observed with low, high or unclear bias risk or applicability concern for each domain, were summarized in a standardized table and figure recommended by the QUADADS-2 official website.

### Data synthesis and statistical analysis

The analysis of diagnostic performance was carried out on both of the per-patient and the per-vessel or per-lesion basis. Using a bivariate model, pooled estimates of sensitivity, specificity, PLR, NLR, DOR, diagnostic score and AUSROC with the corresponding 95% CI were calculated^38^. To be notable, sensitivity, specificity, DOR and AUSROC were regarded as the major outcome measures. Two-tailed P-value of less than 0.05 was considered to be significant.

To estimate a patient’s probability of having a disease of interest after the FFR_CT_ test, Fagan’s nomogram analysis was conducted^39^. Briefly, the plot comprises a vertical axis on the left with fixed pre-test probability, an axis in the middle signifying the likelihood ratio (to what extent the index test would raise or lower the probability of having the disease), and a vertical axis on the right standing for the post-test probability, which indicates the patient’s probability of having the disease (or having the positive result of reference standard test) after the index test result was known.

A Cochrane-Q test was used to assess heterogeneity^40^. Inconsistency index, *I*^*2*^, quantifies the magnitude of heterogeneity as a percentile, with a cut-off valve of *I*^*2*^ at 50% being defined statistically significant heterogeneity. Meta-regression analysis was performed to explore the possible sources of heterogeneity in terms of publication date, study design, sample size, age, gender, diabetes, CT scanner type, time period between FFR_CT_ and FFR, software for FFR_CT_ calculation and cut-off value for FFR_CT_ and FFR. Other possible sources of heterogeneity were not included as a result of data insufficiency in at least one study.

Diagnostic threshold effects were identified by visual evaluation of SROC plots at first and then by Spearman correlation analysis^40^. And publication bias was inspected by Deeks funnel plot, a scatter plot of the inverse of the square root of the effective sample size (1/root (ESS)) against the ln (DOR)^41^.

Data synthesis and most statistical analyses were performed by STATA software version 12.0 (College Station, TX, USA) except the Meta-regression analysis by Meta-Disc version 1.4 (Clinical Biostatistics Unit, Hospital Ramon y Cajal, Madrid, Spain).

## Additional Information

**How to cite this article**: Wu, W. *et al*. Noninvasive fractional flow reserve derived from coronary computed tomography angiography for identification of ischemic lesions: a systematic review and meta-analysis. *Sci. Rep*. **6**, 29409; doi: 10.1038/srep29409 (2016).

## Supplementary Material

Supplementary Information

## Figures and Tables

**Table 1:** Characteristics of included studies and patients’ baseline demographics.

Study	DISCOVER-FLOW^11^	DeFACTO^12^	NXT^15^	Kim *et al*.^13^	Renker *et al*.^14^	Coenen *et al*.^19^	Geer *et al*.^20^
Year	2011	2012	2014	2014	2014	2015	2015
Design	Prospective multicenter	Prospective multicenter	Prospective multicenter	Prospective multicenter	Retrospective single-center	Retrospective single-center	Retrospective single-center
Patients	103	252	254	44	53	106	21
Vessels or lesions	159	408	484	48	67	189	23
Age, year	62.7 ± 8.5	62.9 ± 8.7	64 ± 10	65.0 ± 9.1	61.2 ± 12.0	61.4 ± 9.2	60 ± 7.1
Male (%)	74 (72)	178 (70.6)	162 (64)	35 (80)	34 (64)	82 (77)	NA
Smoking (%)	24 (36)	44 (17.5)	46 (18)	NA	8 (14)	26 (25)	12 (57)
BMI, kg/m^2^	25.8 ± 3.5	NA	26 ± 3	24.4 ± 2.6	28.9 ± 6.5	27.2 ± 4.0	27.6 ± 5.3
Diabetes (%)	26 (26)	53 (21.2)	58 (23)	13 (29)	18 (32)	20 (19)	3 (14)
Prior MI (%)	17 (17)	15 (6.0)	5 (2)	5 (10)	NA	14 (13)	NA
Prior PCI (%)	16 (16)	16 (6.3)	0 (0)	NA	9 (16)	11 (10)	0 (0)
Prior CABG (%)	0 (0)	0 (0)	0 (0)	NA	0 (0)	0 (0)	0 (0)
Hypertension (%)	67 (65)	179 (71.2)	174 (69)	36 (81)	31 (54)	63 (59)	NA
Hyperlipidemia (%)	67 (65)	201 (79.8)	200 (79)	28 (63)	31 (54)	63 (59)	NA
Family history of CAD (%)	NA	50 (19.9)	NA	NA	NA	51 (48)	NA
CT scanner	64 or higher slices	64 or higher slices	64 or higher slices	64 or higher slices	DSCT (64 or 128 slices)	DSCT (64 or 128 slices)	DSCT
Interval between FFR and FFR_CT_, days	2.3 (0–26)	15.5 (5–33)	18 (1–55)	12 (2–40)	≤90	<50	49 (4–106)
Software of FFR_CT_ computation	HeartFlow V1.0	HeartFlow V1.2	HeartFlow V1.4	HeartFlow	Siemens cFFR V1.4	Siemens cFFR V1.4	Siemens cFFR V1.4 & V1.7
Cut-off value of FFR_CT_	≤0.80	≤0.80	≤0.80	≤0.80	<0.80	≤0.80	≤0.80
Cut-off value of FFR	≤0.80	≤0.80	≤0.80	≤0.80	<0.80	≤0.80	≤0.80
TP, per-patient	50	116	69	NA	15	NA	5
FP, per-patient	9	56	37	NA	6	NA	3
FN, per-patient	4	13	11	NA	1	NA	1
TN, per-patient	40	67	137	NA	31	NA	12
TP, per-vessel or lesion	51	121	84	29	17	70	5
FP, per-vessel or lesion	18	96	51	6	7	38	4
FN, per-vessel or lesion	7	30	16	5	3	10	1
TN, per-vessel or lesion	83	160	333	8	40	71	13

Abbreviations: BMI: body mass index; CABG: coronary artery bypass surgery; CAD: coronary artery disease; CT: computed tomography; DSCT: dual source computed tomography; FFR: fractional flow reserve; FFR_CT_: fractional flow reserve derived from computed tomography; FN: false negative; FP: false positive; MI: myocardial infarction; NA: not available; PCI: percutaneous coronary intervention; TN: true negative; TP: true positive.

**Table 2:** Summary table of review authors’ ratings of risk of bias and applicability concerns for each study.

Study	Risk of bias				Applicability concerns		
	Patient selection	Index test	Reference standard	Flow and timing	Patient selection	Index test	Reference standard
DISCOVER-FLOW^4^	⊕	⊕	⊕	⊕	⊕	⊕	⊕
DeFACTO^5^	⊕	⊕	⊕	⊕	⊕	⊕	⊕
NXT^8^	⊕	⊕	⊕	⊕	⊕	⊕	⊕
Kim *et al*.^6^	⊕	⊕	⊕	⊕	⊕	⊕	⊕
Renker *et al*.^7^	⊕	⊕	⊕	⊕	⊕	⊕	⊕
Coenen *et al*.^12^	⊕	⊕	⊕	⊕	⊕	⊕	⊕
Geer *et al*.^13^	⊕	⊕	⊕	⊕	⊕	⊕	⊕

⊕ Low Risk.

⊖ High Risk.

? Unclear Risk.

**Table 3:** Pooled diagnostic performances of FFR_CT_ at the per-patient level and at the per-vessel or per-lesion level.

	Per-patient level	Per-vessel or per-lesion level
Number of included studies	5	7
Number of subjects	833	1377
Sensitivity	0.89 (0.85–0.93)*	0.84 (0.80–0.87)
Specificity	0.76 (0.64–0.84)	0.76 (0.67–0.83)
Positive likelihood ratio	3.68 (2.41–5.61)	3.51 (2.44–5.03)
Negative likelihood ratio	0.14 (0.09–0.21)	0.21 (0.16–0.27)
Diagnostic odds ratio	26.21 (13.14–52.28)	16.87 (9.41–30.25)
Diagnostic score	3.27 (2.58–3.96)	2.83 (2.24–3.41)
AUSROC	0.90 (0.87–0.92)	0.86 (0.83–0.89)

Abbreviations: AUSROC: area of summary receiver operating curve.

^*^Numbers in parentheses are 95% confidence intervals.
