# Supplementary material for: Noninvasive fractional flow reserve derived from coronary computed tomography angiography for identification of ischemic lesions: a systematic review and meta-analysis
Source: Sci Rep. 2016 Jul 5;6:29409. doi: 10.1038/srep29409 (PMC4932511; doi:10.1038/srep29409)
Supplement: Supplementary Information [file srep29409-s1.pdf]

Noninvasive fractional flow reserve derived from coronary computed tomography angiography for identification of ischemic lesions: a systematic review and meta-analysis

Wen Wu[1, +], Dao-Rong Pan[1, +], Nicolas Foin[2, +], Si Pang[3], Peng Ye[1], Niels Holm[4], Xiao-Min Ren[1], Jie Luo[1], Aravinda Nanjundappa[5] and Shao-Liang Chen[1, *]

[1]Department of Cardiology, Nanjing First Hospital, Nanjing Medical University, Nanjing, 210006, P.R. China
[2]National Heart Research Institute, National Heart Centre Singapore, 169609, Singapore
[3]Department of Cardiology, Zhongda Hospital, Medical School of Southeast University, Nanjing 210009, China
[4]Department of Cardiology, Aarhus University Hospital, Aarhus, 8000, Denmark
[5]Division of Vascular Surgery, West Virginia University, Morgantown, 25304, USA.
*Correspondence and requests for materials should be addressed to S.-L.C. (email: chmengx@sina.com).
[+]These authors contributed equally to this work.

Study	Inclusion	Exclusion
DISCOVER-FLOW	CAD (suspected or known), stenosis > 50% in coronary CTA in a major coronary artery (≥ 2.0 mm diameter), age > 18 years, and undergoing clinically indicated ICA with FFR.	Unable to provide informed consent; Prior CABG; serum creatinine level > 1.5 mg/dl; non-cardiac illness with life expectancy < 2 years; pregnant state; allergy to iodinated contrast; significant arrhythmia; heart rate ≥ 100 beats/min; systolic blood pressure ≤ 90 mm Hg; contraindication to beta blockers, nitroglycerin or adenosine; Canadian Cardiovascular Society class IV angina; or non-evaluable CCTA as determined by the CCTA core laboratory.
DeFACTO	CAD (suspected), no intervening coronary event.	History of CABG; prior PCI with suspected in-stent restenosis; contraindication to adenosine; suspicion of or recent ACS; complex congenital heart disease; prior pacemaker or defibrillator; prosthetic heart valve; significant arrhythmia; serum creatinine level > 1.5 mg/dl; BMI > 35 kg/m^2; allergy to iodinated contrast; pregnant state; evidence of active clinical instability or life threatening disease; or inability to adhere to study procedures.
NXT	Coronary CTA performed < 60 days before scheduled non-emergent ICA.	Previous PCI or CABG; suspected ACS; previous MI < 30 days before procedure; BMI > 35 kg/m^2; contraindication to beta blockers, nitroglycerin or adenosine
Kim et al	CAD (suspected or known), age ≥ 18 years, stenosis ≥ 50% in coronary CTA in a major coronary artery (≥ 2.0 mm diameter).	Reaching upper threshold of qualitative coronary calcification, heart rate, or BMI; any intervening coronary event within 45 days.
Renker et al	CAD (suspected or known).	Time between procedures exceeding 3 months, inter-procedural major adverse cardiac events (cardiac death, nonfatal MI, or revascularization), severely reduced left ventricular function, previous CABG, stent placement for lesion of interest, bifurcation stenosis types D to G, severe stenosis of the proximal LM and/or the proximal RCA, chronic total occlusion.
Coenen et al	Suspected or known CAD, no history of CABG or PCI in the vessel of interest.	Cardiac events between coronary CTA and invasive FFR procedure, non-interpretable coronary CT angiography image quality, incomplete coronary CT angiography coverage; an Agatston coronary artery calcium score > 2000.
Geer et al	CAD (referred to ICA within ≤ 120 days of the coronary CTA) in RCA, left anterior descending artery, or circumflex artery.	Previous CABG or PCI, ostial stenosis; No pressure guidewire image; Suboptimal image quality; Missing thin section data; Obvious discrepancy invasive FFR angiograph.

Supplementary Table 1. Inclusion and exclusion criteria for study population. Abbreviations: ACS: acute coronary syndrome; BMI: body mass index; CABG: coronary artery bypass surgery; CAD: coronary artery disease; CT: computed tomography; CTA: computed tomography angiography; FFR: fractional flow reserve; ICA: invasive coronary artery angiography; LM: left main coronary artery; MI: myocardial infarction; PCI: percutaneous coronary intervention; RCA: right coronary artery.

	Per-patient level				Per-vessel or per-lesion level			
	Univariate model		Bivariate model		Univariate model		Bivariate model	
Number of included studies	4	5	4	5	5	7	5	7
Number of subjects	662	833	662	833	1165	1377	1165	1377
Sensitivity	0.90 (0.85-0.93) *	0.90 (0.85-0.93)	0.90 (0.85-0.93)	0.89 (0.85-0.93)	0.83 (0.79-0.87)	0.84 (0.80-0.87)	0.84 (0.79-0.88)	0.84 (0.80-0.87)
Specificity	0.72 (0.67-0.76)	0.72 (0.67-0.77)	0.75 (0.62-0.85)	0.76 (0.64-0.84)	0.78 (0.75-0.81)	0.76 (0.73-0.79)	0.78 (0.67-0.86)	0.76 (0.67-0.83)
Diagnostic odds ratio	24.34 (10.84-54.65)	23.22 (11.43-47.19)	25.87 (12.27-54.53)	26.21 (13.14-52.28)	17.89 (7.28-43.98)	16.76 (8.52-32.95)	18.22 (8.73-38.02)	16.87(9.41-30.25)
AUSROC	0.94 (0.88-1.00)	0.93 (0.88-0.97)	0.90 (0.87-0.92)	0.90 (0.87-0.921387)	0.91 (0.95-0.87)	0.91 (0.87-0.95)	0.87(0.83-0.89)	0.86 (0.83-0.89)

Supplementary Table 2. Pooled diagnostic performances of FFR_{CT} at the per-patient level and at the per-vessel or per-lesion level except two recent studies (Coenen et al and Geer et al) using two different pooling statistical methods. Univariate model includes a fixed-effects model and a random-effects model. Data with heterogeneity (defined as an I^2 value of more than 50%) were combined using the random-effects model (DerSimonian-Laird model) while data without heterogeneity were synthesized by the fixed-effects model (Mantel-Haenszel model). Abbreviations: AUSROC: area of summary receiver operating curve; *: numbers in parentheses are 95% confidence intervals.

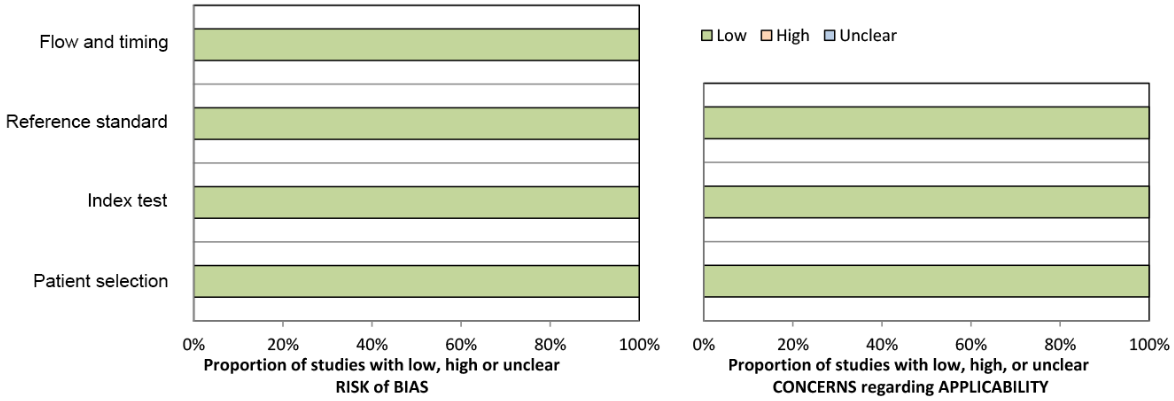

Supplementary Figure 1. Cumulative bar plot of risk of bias and applicability concerns across all studies.
